# Mucin 1 as a Molecular Target of a Novel Diisoquinoline Derivative Combined with Anti-MUC1 Antibody in AGS Gastric Cancer Cells

**DOI:** 10.3390/molecules26216504

**Published:** 2021-10-28

**Authors:** Agnieszka Gornowicz, Wojciech Szymanowski, Krzysztof Bielawski, Zbigniew Kałuża, Olga Michalak, Anna Bielawska

**Affiliations:** 1 Department of Biotechnology, Medical University of Bialystok, 15-222 Bialystok, Poland; wojciech.szymanowski@umb.edu.pl (W.S.); anna.bielawska@umb.edu.pl (A.B.); 2 Department of Synthesis and Technology of Drugs, Medical University of Bialystok, 15-222 Bialystok, Poland; kbiel@umb.edu.pl; 3 Institute of Organic Chemistry, Polish Academy of Sciences, 01-224 Warsaw, Poland; zbigniew.kaluza@icho.edu.pl; 4 Department of Pharmacy, Cosmetic Chemistry and Biotechnology, Team of Chemistry, Łukasiewicz Research Network—Industrial Chemistry Institute, 01-793 Warsaw, Poland; olga.michalak@ichp.pl

**Keywords:** MUC1, diisoquinoline derivative, apoptosis, autophagy, gastric cancer

## Abstract

Background: The aim of the study was to examine the molecular mechanism of the anticancer action of a monoclonal antibody against MUC1 and a diisoquinoline derivative (OM-86II) in human gastric cancer cells. Methods: The cell viability was measured by the MTT assay. The disruption of mitochondrial membrane potential and activity of caspase-8 and caspase-9 was performed by flow cytometry. Fluorescent microscopy was used to confirm the proapoptotic effect of compounds. LC3A, LC3B and Beclin-1 concentrations were analyzed to check the influence of the compounds on induction of autophagy. ELISA assessments were performed to measure the concentration of mTOR, sICAM1, MMP-2, MMP-9 and pro-apoptotic Bax. Results: The anti-MUC1 antibody with the diisoquinoline derivative (OM-86II) significantly reduced gastric cancer cells’ viability. This was accompanied by an increase in caspase-8 and caspase-9 activity as well as high concentrations of pro-apoptotic Bax. We also proved that the anti-MUC1 antibody with OM-86II decreased the concentrations of MMP-9, sICAM1 and mTOR in gastric cancer cells. After 48 h of incubation with such a combination, we observed higher levels of the crucial component of autophagosomes (LC3) and Beclin-1. Conclusions: Our study proved that the anti-MUC1 antibody sensitizes human gastric cancer cells to the novel diisoquinoline derivative (OM-86II) via induction of apoptosis and autophagy, and inhibition of selected proteins such as mTOR, sICAM1 and MMP-9.

## 1. Introduction

Mucin 1 (MUC1) is a member of the mucin family (MUC1–MUC21) and plays different roles in normal and gastric cancer cells. The MUC1 extracellular domain acts as a protective barrier during bacterial invasion with Helicobacter pylori and Campylobacter jejuni [1,2]. However, MUC1 also plays a relevant role in the development and progression of gastric cancer [3]. A number of reports have demonstrated a strong correlation between increased MUC1 expression and worse prognosis in patients with gastric cancer [4,5]. Its cancer-promoting function is associated with failure of apoptosis induction by the activation of anti-apoptotic Bcl-xL [6]. MUC1 also inhibits apoptosis by attenuating the nuclear translocation of non-receptor c-Abl tyrosine kinase. As a result, gastric cancer cells demonstrate resistance to anticancer agents. MUC1 is also responsible for the p53 gene transcription, as well as increased activation of the PI3K→AKT and RAS→RAF→MEK→ERK pathways [7]. The interaction between intercellular adhesion molecule-1 (ICAM-1) and mucin 1 facilitates the migration of cancer cells through the vessel walls and favors metastasis and cancer progression [8]. Most of the preclinical and clinical studies, in which agents were able to block MUC1 function, were performed on breast cancer cells. However, MUC1 might also be a promising target in other carcinomas characterized by its overexpression.

Aberrant activation of the PI3K/AKT/mTOR signaling pathway is demonstrated in many human cancers and promotes tumor progression. It plays a key role in the metabolism, growth, migration, invasion and survival of cancer cells [9,10]. Significant advances in identifying and understanding abnormalities in the PI3K/AKT/mTOR pathway have enabled the development of new targeted molecules that offer hope for a more effective anticancer therapy. Currently, two anticancer drugs that are inhibitors of mTOR kinase are used in medicine: everolimus and temsirolimus [11]. They have been registered by the FDA and EMA for the treatment of a variety of cancers, including advanced renal cell carcinoma, HER2-negative breast cancer and pancreatic tumors. In clinical trials, PI3K/mTOR inhibitors (PQR309 and PKI-587/PF-05212384—Gedatolisib) as well as mTOR kinase inhibitors (AZD2014, TAK-228 and CC-223) have been investigated [12]. 

Numerous studies confirm that invasion and metastasis correlate with increased expression of specific metalloproteinases (MMPs) in cancer cells. The ability to break down the basement membrane ECM proteins is possessed in particular by metalloproteinase-2 (MMP-2) and metalloproteinase-9 (MMP-9) [13]. Immunohistochemical studies indicate overexpression of MMP-2 and MMP-9 in 78% and 70% of all tested stomach cancer samples, respectively [14]. The involvement of metalloproteinases (MMPs) in metastasis has been proven in in vitro studies and in vivo models [15]. Understanding the mechanisms of metalloproteinase activity regulation in various cellular processes, such as apoptosis, angiogenesis, invasion, metastasis or immune response, is extremely important for early diagnosis and development of better treatments for stomach cancers. 

Cathepsin B is a member of the cysteine proteases, and its high activity is observed in gastric carcinoma. The higher levels of cathepsin B are also demonstrated in the urine and serum from patients with gastric carcinoma [16]. The increased expression of cathepsin B is associated with more aggressive potential and plays a role in gastric cancer invasion, so it may be a promising target in anticancer treatment [17]. Czyżewska et al. found a strong, statistically significant association between the expression of MMP-9 and cathepsin B in the main tumor mass and lymph node involvement in advanced gastric carcinoma, but not with patients’ postoperative survival [18].

Autophagy is a highly regulated process and is categorized into three forms: macro-autophagy, micro-autophagy and chaperone-mediated autophagy (CMA) [19,20]. The formation of autophagosomes begins with the generation of a double lipid membrane, which matures from an early phagophore into a mature autophagosome containing lipidated ATG microtubule-associated proteins 1A/1B light-chain 3 proteins (MAP1LC3 proteins, LC3 proteins). In humans, we can distinguish three members of the LC3 family: LC3A, LC3B and LC3C. LC3B is a well-known marker of autophagy [20]. Dysregulation of this process is connected with many diseases, such as type II diabetes, infectious diseases, neurodegenerative diseases, cardiomyopathy as well as cancer [21,22,23,24,25]. 

Recent studies also demonstrated that autophagy is engaged in gastric cancer progression and metastasis [26,27,28]. There is a link between autophagy and *Helicobacter pylori* infection in gastric cancer [26]. It was proven that inhibition of autophagy by 3-methyladenine favors the intracellular replication and survival of *H. pylori*, while the autophagy activator rapamycin promotes bacterial clearance in gastric epithelial cells [29]. Researchers are still investigating the autophagy inhibitors or activators that represent a new strategy for the treatment of human cancer.

The aim of this study was to characterize the molecular mechanisms of action induced by the monoclonal antibody against MUC1 in combination with the novel diisoquinoline derivative (OM-86II) in human gastric cancer cells. We analyzed the combinatorial effect of such a treatment on viability, DNA biosynthesis, apoptosis induction and decrease in mitochondrial membrane potential. The influence of the tested compounds on the induction of programmed cell death was confirmed by dual acridine orange/ethidium bromide staining. The activity of crucial caspases (-8 and -9) engaged in molecular extrinsic and intrinsic apoptotic pathways was also demonstrated. Finally, a key element of the study was to explain the effect of the anti-MUC1 antibody with OM-86II on the concentrations of selected proteins, such as: mTOR, sICAM1, MMP-2, MMP-9, Bax, LC3A, LC3B, Beclin-1 and cathepsin B.

## 2. Results

### 2.1. Novel Diisoquinoline Derivative with Anti-MUC1 Antibody Inhibits the Growth and Proliferation of Human Gastric Cancer Cells

**Figure 1:**
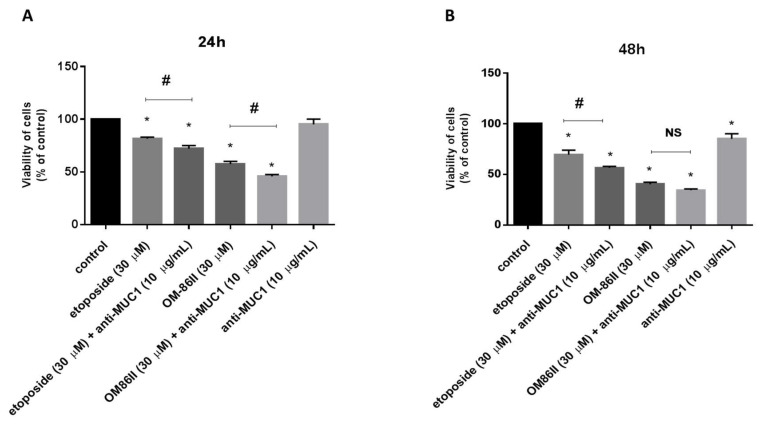
The effect of anti-MUC1 (10 μg/mL), OM-86II (30 μM), OM-86II + anti-MUC1 (30 μM + 10 μg/mL), etoposide (30 μM) and etoposide + anti-MUC1 (30 μM + 10 μg/mL) on the viability of AGS gastric cancer cells after 24 h (**A**) and 48 h (**B**) of incubation. Mean ± SD from three independent experiments (*n* = 3), performed in duplicate, are presented. * *p* < 0.05 vs. control group; # *p* < 0.05. MUC1, mucin-1; OM-86II, octahydropyrazin[2,1-a:5,4-a′]diisoquinoline derivative; NS, not significant.

**Figure 2:**
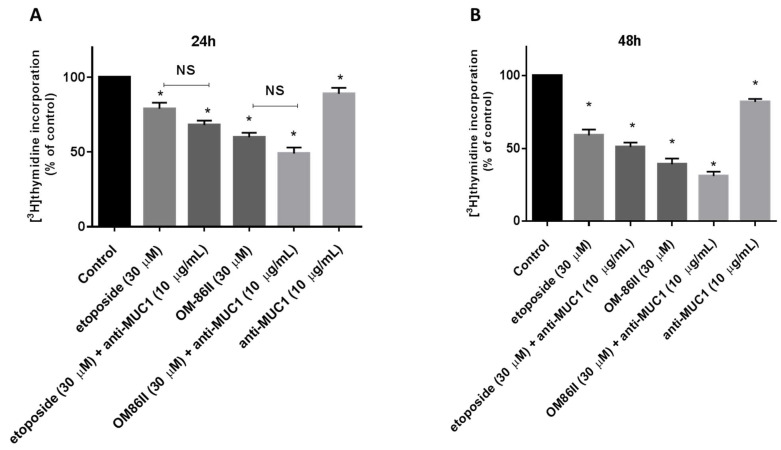
The effect of anti-MUC1 (10 μg/mL), OM-86II (30 μM), OM-86II + anti-MUC1 (30 μM + 10 μg/mL), etoposide (30 μM) and etoposide + anti-MUC1 (30 μM + 10 μg/mL) on DNA biosynthesis in cultured AGS cells after 24 h (**A**) and 48 h (**B**) of incubation, as measured by inhibition of [^3^H]-thymidine incorporation into DNA. Mean ± SD from three independent experiments (*n* = 3), performed in duplicate, are presented. * *p* < 0.05 vs. control group; MUC1, mucin-1; OM-86II, octahydropyrazin[2,1-a:5,4-a′]diisoquinoline derivative; NS, not significant.

The preliminary study proved that IC_50_ values for OM-86II, etoposide and anti-MUC1 were 42 µM, 80 µM and 26 µg/mL, respectively. We checked the effect of the tested compounds in lower doses than IC_50_ (OM-86II, etoposide, anti-MUC1 antibody, OM-86II + anti-MUC1, etoposide + anti-MUC1) on viability and DNA biosynthesis in gastric cancer cells after 24 and 48 h of incubation (Figure 1 and Figure 2). Our studies revealed that less cytotoxicity was caused by the anti-MUC1 antibody, which reduced the cell viability to 95% after 24 h of incubation and to 85% after 48 h of incubation. The novel compound OM-86II was more cytotoxic than the reference etoposide. It decreased the viability of cancer cells to 57.5% and 40.2% after 24 and 48 h of incubation respectively, whereas the etoposide inhibited the viability of cells to 81.5% and 69.4%, respectively. We detected 72% and 56.4% of viable cells after treatment with the anti-MUC1 antibody together with etoposide. However, the combination of the anti-MUC1 antibody together with OM-86II was the most lethal, which decreased the number of viable cells to 45.8% and 34.3% after 24 and 48 h of incubation (Figure 1A,B).

The antiproliferative properties were proven by checking the influence of the tested compounds on DNA biosynthesis in the analyzed gastric cancer cells. After 24 h of incubation, we observed that OM-86II used together with anti-MUC1 reduced DNA biosynthesis to 49%, whereas the weakest effect was demonstrated after incubation with the anti-MUC1 antibody, which inhibited the process up to 89% (Figure 2A). The longer exposition of gastric cancer cells to the tested compounds led to higher reduction of DNA biosynthesis (Figure 2B). The combination of OM-86II with the anti-MUC1 antibody decreased DNA biosynthesis to 31%, and the antiproliferative effect was stronger than that evoked by etoposide (59%), OM-86II (39%), anti-MUC1 (82%) and the combination of etoposide used with anti-MUC1 (51%) (Figure 2B).

### 2.2. Novel Diisoquinoline Derivative with Anti-MUC1 Antibody Possesses Pro-Apoptotic Activity

**Figure 3:**
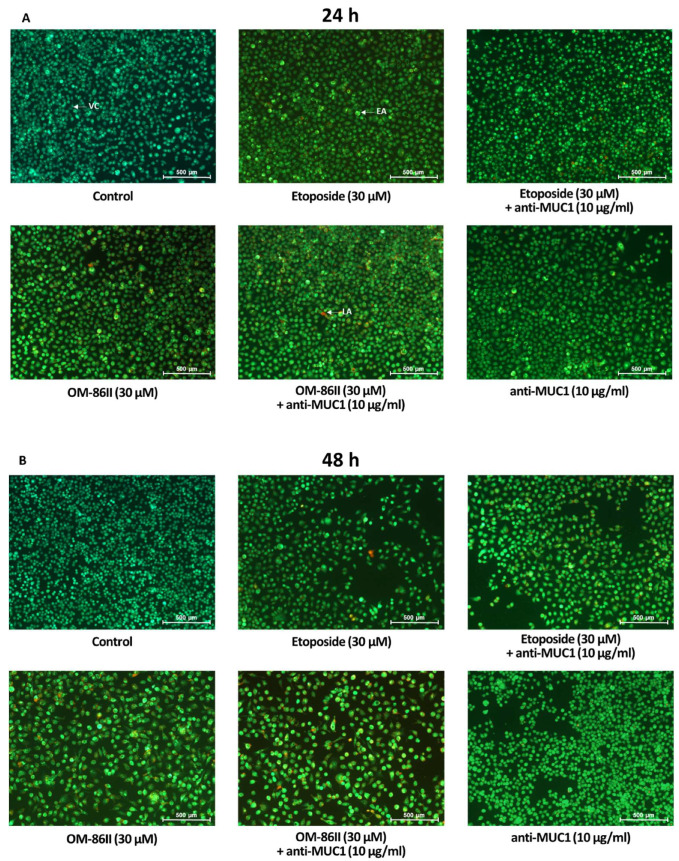
The influence of anti-MUC1 (10 μg/mL), OM-86II (30 μM), OM-86II + anti-MUC1 (30 μM + 10 μg/mL), etoposide (30 μM) and etoposide + anti-MUC1 (30 μM + 10 μg/mL) on induction of apoptosis in human AGS cells after 24 h (**A**) and 48 h (**B**) of incubation. The evaluation was performed by a fluorescent microscopy after acridine orange and ethidium bromide staining.

**Figure 4:**
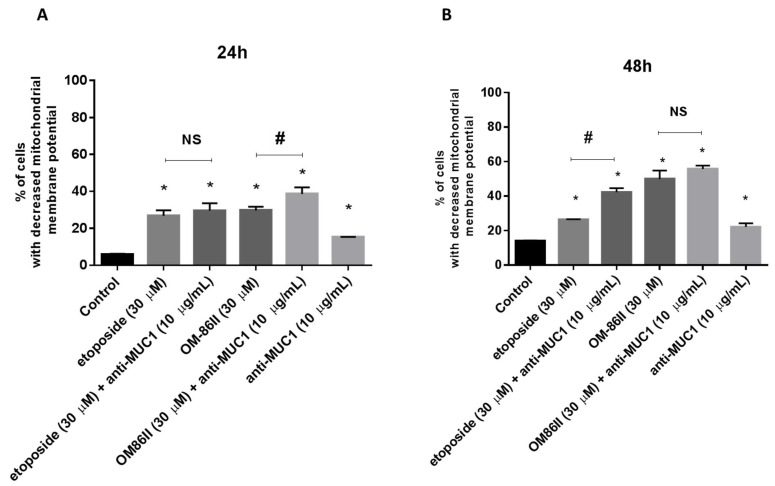
Fluorescence of AGS gastric cancer cells treated for 24 h (**A**) and 48 h (**B**) with anti-MUC1 (10 μg/mL), OM-86II (30 μM), OM-86II + anti-MUC1 (30 μM + 10 μg/mL), etoposide (30 μM) and etoposide + anti-MUC1 (30 μM + 10 μg/mL) incubated with mitochondrial membrane potential probe JC-1. X- and y-axes are green and red fluorescence, respectively. Mean percentage values from three independent experiments (*n* = 3), performed in duplicate, are presented. * *p* < 0.05 vs. control group; # *p* < 0.05. MUC1, mucin-1; OM-86II, octahydropyrazin[2,1-a:5,4-a′]diisoquinoline derivative; NS, not significant.

AGS cancer cells were exposed to different concentrations of the tested compounds for 24 and 48 h. After the incubation, the proapoptotic effect of the analyzed compounds was checked using dual acridine orange/ethidium bromide fluorescent staining. Control cells (untreated) were identified as green fluorescence, early apoptotic cells as bright green fluorescence and late apoptotic cells were presented by a reddish-orange color. The results of the staining are presented in Figure 3A,B. The combination of the anti-MUC1 antibody with the diisoquinoline derivative (OM-86II) was the most efficient strategy that led to induction of apoptosis. We could observe the changes in the morphology of cells characteristic for early and late apoptosis. The combination of compounds was more effective than monotherapy based on the anti-MUC1 antibody, etoposide or OM-86II. Although, we proved that a combination of anti-MUC1 with the novel diisoquinoline derivative caused a decrease in mitochondrial membrane potential. We detected 38.6% and 55.7% of cells with decreased MMP after 24 and 48 h of incubation, respectively (Figure 4A,B). Taking into account the treatment of cells with a single compound, we proved that compound OM-86II decreased MMP more efficiently than etoposide or anti-MUC1. We showed that 29.7% and 49.9% of analyzed gastric cancer cells had decreased MMP after 24 and 48 h of incubation, respectively.

### 2.3. Novel Diisoquinoline Derivative with Anti-MUC1 Antibody Increases Caspase-8 and Caspase-9 Activity

**Figure 5:**
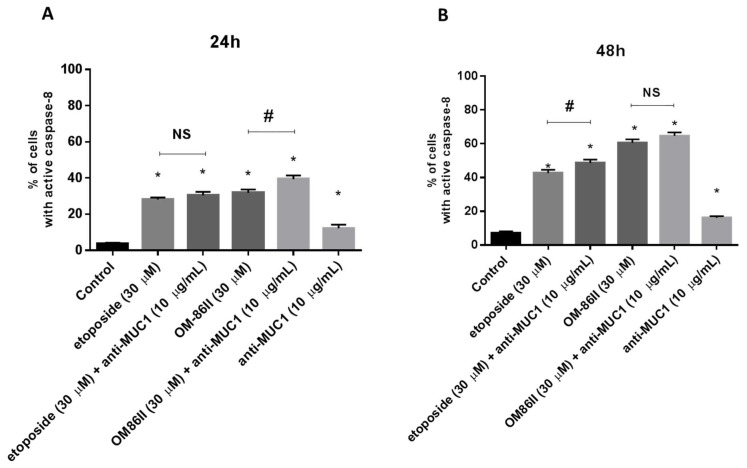
Caspase-8 activity in AGS gastric cancer cells treated for 24 h (**A**) and 48 h (**B**) with anti-MUC1 (10 μg/mL), OM-86II (30 μM), OM-86II + anti-MUC1 (30 μM + 10 μg/mL), etoposide (30 μM) and etoposide + anti-MUC1 (30 μM + 10 μg/mL) was demonstrated using flow cytometry. Mean percentage values from three independent experiments (*n* = 3), performed in duplicate, are presented. * *p* < 0.05 vs. control group; # *p* < 0.05. MUC1, mucin-1; OM-86II, octahydropyrazin[2,1-a:5,4-a′]diisoquinoline derivative; NS, not significant.

**Figure 6:**
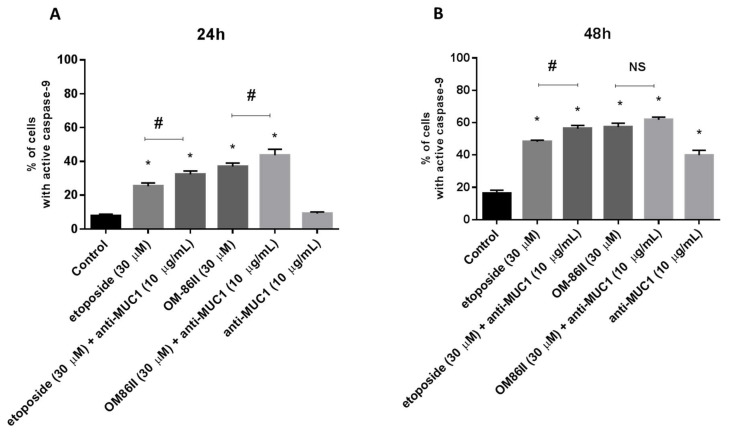
Caspase-9 activity in AGS gastric cancer cells treated for 24 h (**A**) and 48 h (**B**) with anti-MUC1 (10 μg/mL), OM-86II (30 μM), OM-86II + anti-MUC1 (30 μM + 10 μg/mL), etoposide (30 μM) and etoposide + anti-MUC1 (30 μM + 10 μg/mL) was demonstrated using flow cytometry. Mean percentage values from three independent experiments (*n* = 3), performed in duplicate, are presented. * *p* < 0.05 vs. control group; # *p* < 0.05. MUC1, mucin-1; OM-86II, octahydropyrazin[2,1-a:5,4-a′]diisoquinoline derivative; NS, not significant.

Flow cytometry was used to determine caspase-8 and caspase-9 activity in gastric cancer cells after 24 and 48 h of incubation with the tested compounds. We detected 28.1% and 31.8% of cells with active caspase-8 after 24 h of incubation with etoposide and OM-86II. After a further 24 h, we noticed 42.6% and 60.6% of cells with active caspase-8 after exposition to etoposide and OM-86II. We proved that treatment of cells with single compounds also activated caspase-9. After 48 h of incubation, we demonstrated 48.1% and 57.3% of cells with active caspase-9 after treatment with etoposide and OM-86II. The analysis revealed that the combination of the anti-MUC1 antibody with OM-86II activated both caspases in AGS gastric cancer cells. Studies proved that 39.4% of the analyzed population of cells had active caspase-8 and 43.6% had active caspase-9 after 24 h of incubation compared to the control (3.6%, 7.8%). After the next 24 h, the percentage of cells with active caspase-8 and caspase-9 increased in comparison with the untreated cells (7.1%, 16.2%). We noticed 64.7% of cells with active caspase-8 and 61.7% of cells with active caspase-9 after combined treatment with anti-MUC1 and OM-86II. The effect was stronger than the anti-MUC1 antibody used with etoposide, OM-86II, etoposide or the anti-MUC1 antibody (Figure 5 and Figure 6).

### 2.4. Novel Diisoquinoline Derivative with Anti-MUC1 Antibody Significantly Decreased the Concentrations of mTOR, sICAM1, MMP-9 and Bax after 48 h of Incubation

AGS cells were exposed to different concentrations of agents used alone (OM-86II, etoposide, anti-MUC1 antibody) and in combination with the anti-MUC1 antibody for 24 and 48 h. The concentrations of mTOR, sICAM1, MMP-9 and MMP-2 were analyzed.

**Figure 7:**
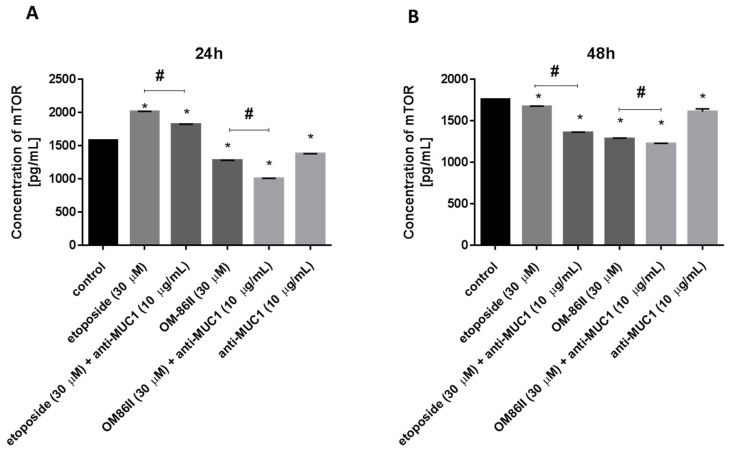
The concentration of mTOR in human gastric cancer AGS cells after 24 h (**A**) and 48 h (**B**) of incubation with anti-MUC1 (10 μg/mL), OM-86II (30 μM), OM-86II + anti-MUC1 (30 μM + 10 μg/mL), etoposide (30 μM) and etoposide + anti-MUC1 (30 μM + 10 μg/mL). Data are presented in ng/mL. * *p* < 0.05 vs. control group; # *p* < 0.05. MUC1, mucin-1; OM-86II, octahydropyrazin[2,1-a:5,4-a′]diisoquinoline derivative.

After 24 h of incubation, etoposide as well as etoposide with the anti-MUC1 antibody led to an increase of mTOR concentration in comparison with untreated cells. We detected 2015 pg/mL after incubation with etoposide and 1823 pg/mL after treatment with etoposide and the anti-MUC1 antibody, respectively. Anti-MUC1, OM-86II and OM-86II in combination with anti-MUC1 led to a decrease in mTOR concentration. The highest decrease was observed after 24 h of incubation with OM-86II with the anti-MUC1 antibody, where we detected 1005 pg/mL of mTOR in cell lysates, whereas in untreated cells, the concentration of mTOR was 1582 pg/mL. However, after another 24 h, we demonstrated that all the examined compounds used alone and in combination with the anti-MUC1 antibody decreased the mTOR concentration. The most significant decrease was also determined after 48 h of incubation with a combination of OM-86II and anti-MUC1 (Figure 7A,B).

**Figure 8:**
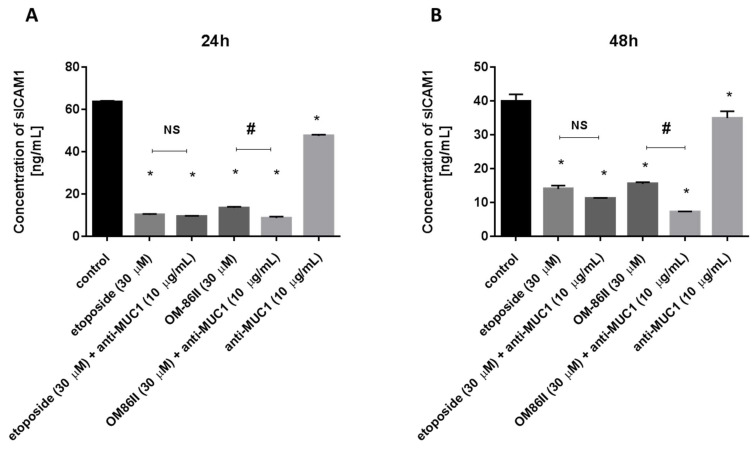
The concentration of sICAM1 in human gastric cancer AGS cells after 24 h (**A**) and 48 h (**B**) of incubation with anti-MUC1 (10 μg/mL), OM-86II (30 μM), OM-86II + anti-MUC1 (30 μM + 10 μg/mL), etoposide (30 μM) and etoposide + anti-MUC1 (30 μM + 10 μg/mL). Data are presented in ng/mL. * *p* < 0.05 vs. control group; # *p* < 0.05. MUC1, mucin-1; OM-86II, octahydropyrazin[2,1-a:5,4-a′]diisoquinoline derivative; NS, not significant.

We analyzed the sICAM1 concentration after 24 and 48 h of incubation with the tested compounds and we demonstrated that agents used alone and in combination with the monoclonal antibody decreased the sICAM1 concentration in media from the cell culture (Figure 8A,B). The combination of OM-86II or etoposide with anti-MUC1 represented the most effective strategy in decreasing the sICAM1 concentration in comparison with untreated cells and monotherapy. We detected 11.2 pg/mL after 48 h of incubation with etoposide and anti-MUC1, and 7.2 pg/mL after 48 h of incubation with OM-86II with anti-MUC1, whereas in the control sample, the concentration of sICAM1 was 40 pg/mL (Figure 8B).

**Figure 9:**
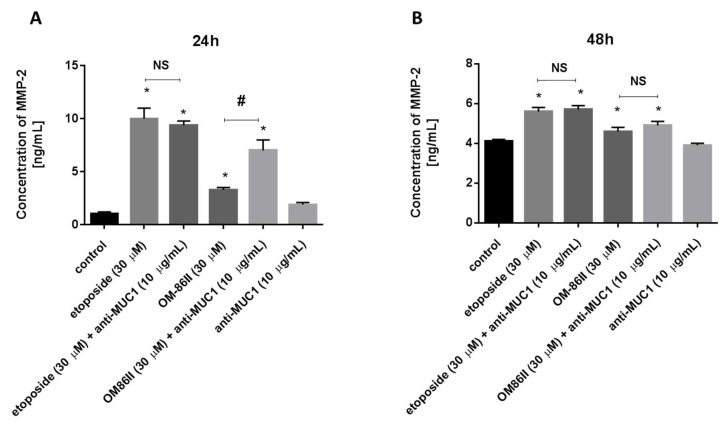
The concentration of MMP-2 in human gastric cancer AGS cells after 24 h (**A**) and 48 h (**B**) of incubation with anti-MUC1 (10 μg/mL), OM-86II (30 μM), OM-86II + anti-MUC1 (30 μM + 10 μg/mL), etoposide (30 μM) and etoposide + anti-MUC1 (30 μM + 10 μg/mL). Data are presented in ng/mL. * *p* < 0.05 vs. control group; # *p* < 0.05. MUC1, mucin-1; OM-86II, octahydropyrazin[2,1-a:5,4-a′]diisoquinoline derivative; NS, not significant.

**Figure 10:**
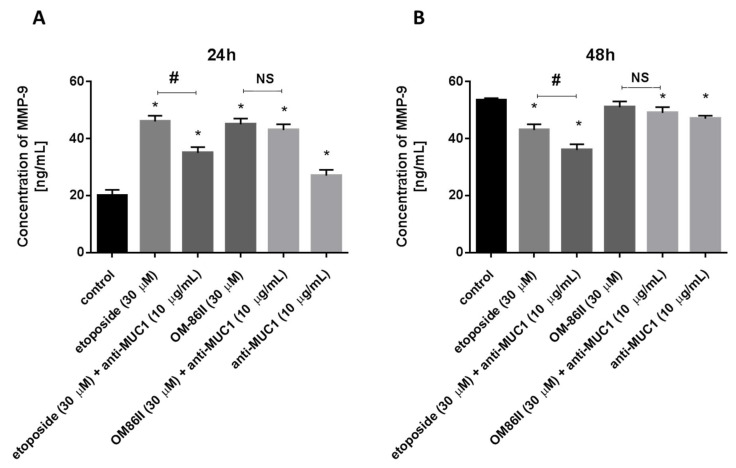
The concentration of MMP-9 in human gastric cancer AGS cells after 24 h (**A**) and 48 h (**B**) of incubation with anti-MUC1 (10 μg/mL), OM-86II (30 μM), OM-86II + anti-MUC1 (30 μM + 10 μg/mL), etoposide (30 μM) and etoposide + anti-MUC1 (30 μM + 10 μg/mL). Data are presented in ng/mL. * *p* < 0.05 vs. control group; # *p* < 0.05. MUC1, mucin-1; OM-86II, octahydropyrazin[2,1-a:5,4-a′]diisoquinoline derivative; NS, not significant.

We further examined the concentrations of matrix metalloproteases-2 and -9 in media after combined treatment and monotherapy. After 24 h of incubation, the results of the ELISA measurement revealed that MMP-9 and MMP-2 levels after treatment with the agents alone and in combination with the anti-MUC1 antibody were significantly higher in the supernatant medium compared to the untreated cells. The results are presented in Figure 9A and Figure 10A. The prolongation of time exposure to 48 h led to the reduction of MMP-9 levels, but not MMP-2, in media from the cell culture (Figure 9B and Figure 10B).

**Figure 11:**
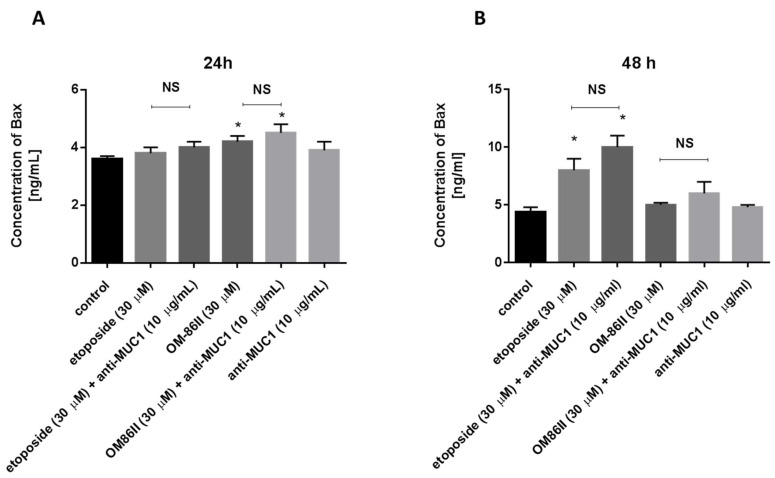
The concentration of pro-apoptotic Bax in human gastric cancer AGS cells after 24 h (**A**) and 48 h (**B**) of incubation with anti-MUC1 (10 μg/mL), OM-86II (30 μM), OM-86II + anti-MUC1 (30 μM + 10 μg/mL), etoposide (30 μM) and etoposide + anti-MUC1 (30 μM + 10 μg/mL). Data are presented in ng/mL. * *p* < 0.05 vs. control group; MUC1, mucin-1; OM-86II, octahydropyrazin[2,1-a:5,4-a′]diisoquinoline derivative; NS, not significant.

Additionally, we checked the concentration of Bax to confirm the proapoptotic potential of the tested compounds and the results obtained by flow cytometry and fluorescence microscopy. All compounds led to an increase in Bax concentration after 24 and 48 h of incubation, which is shown in Figure 11A,B.

### 2.5. Novel Diisoquinoline Derivative with Anti-MUC1 Antibody Increased LC3A and LC3B Concentrations after 48 h of Incubation in Human Gastric Cancer Cells

**Figure 12:**
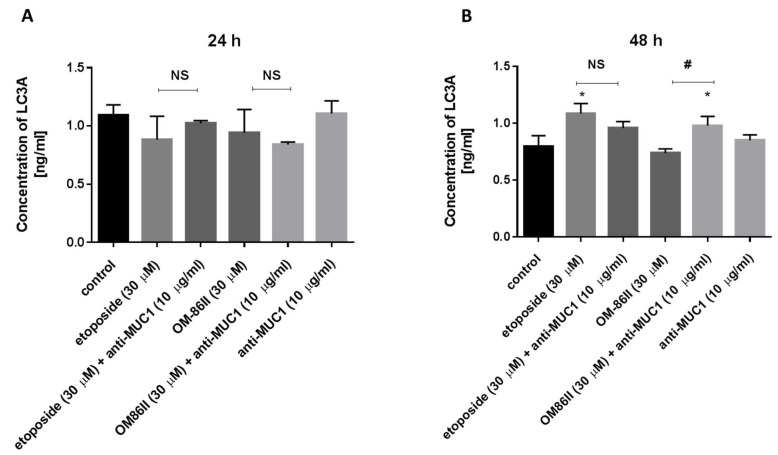
The concentration of LC3A in human gastric cancer AGS cells after 24 h (**A**) and 48 h (**B**) of incubation with anti-MUC1 (10 μg/mL), OM-86II (30 μM), OM-86II + anti-MUC1 (30 μM + 10 μg/mL), etoposide (30 μM) and etoposide + anti-MUC1 (30 μM + 10 μg/mL). Data are presented in ng/mL. * *p* < 0.05 vs. control group; # *p* < 0.05. MUC1, mucin-1; OM-86II, octahydropyrazin[2,1-a:5,4-a′]diisoquinoline derivative; NS, not significant.

**Figure 13:**
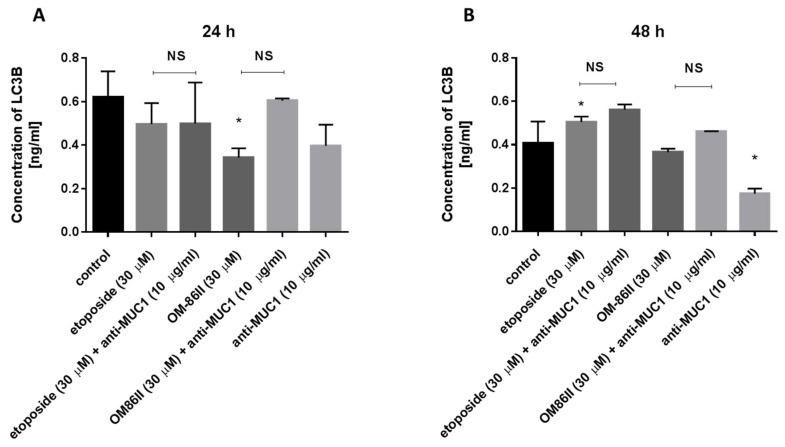
The concentration of LC3B in human gastric cancer AGS cells after 24 h (**A**) and 48 h (**B**) of incubation with anti-MUC1 (10 μg/mL), OM-86II (30 μM), OM-86II + anti-MUC1 (30 μM + 10 μg/mL), etoposide (30 μM) and etoposide + anti-MUC1 (30 μM + 10 μg/mL). Data are presented in ng/mL. * *p* < 0.05 vs. control group; MUC1, mucin-1; OM-86II, octahydropyrazin[2,1-a:5,4-a′]diisoquinoline derivative; NS, not significant.

Moreover, the concentrations of microtubule-associated protein 1 light-chain 3A (LC3A) and microtubule-associated protein 1 light-chain 3B (LC3B) are also demonstrated in Figure 12 and Figure 13. Our research revealed that all compounds used alone and in combination with the anti-MUC1 antibody did not increase the concentrations of LC3A and LC3B after 24 h of incubation compared to the untreated cells (1091 ng/mL, 0.62 ng/mL). The longer exposition of cells to the combination of compounds (etoposide + anti-MUC1, OM-86II + anti-MUC1) led to an increase of LC3A and LC3B. The concentrations of LC3A were 0.958 ng/mL for etoposide with the anti-MUC1 antibody and 0.981 ng/mL for OM-86II with the anti-MUC1 antibody after 48 h of incubation, in comparison with the control (0.796 ng/mL). The concentrations of LC3B were 0.562 ng/mL for etoposide with the anti-MUC1 antibody and 0.462 ng/mL for OM- 86II with the anti-MUC1 antibody after 48 h of incubation compared to the untreated cells (0.407 ng/mL). The analysis proved that 48 h of incubation with the tested combinations induced autophagy in gastric cancer cells.

### 2.6. Novel Diisoquinoline Derivative with Anti-MUC1 Antibody Increased Beclin-1 Concentration after 48 h of Incubation in Human Gastric Cancer Cells

**Figure 14:**
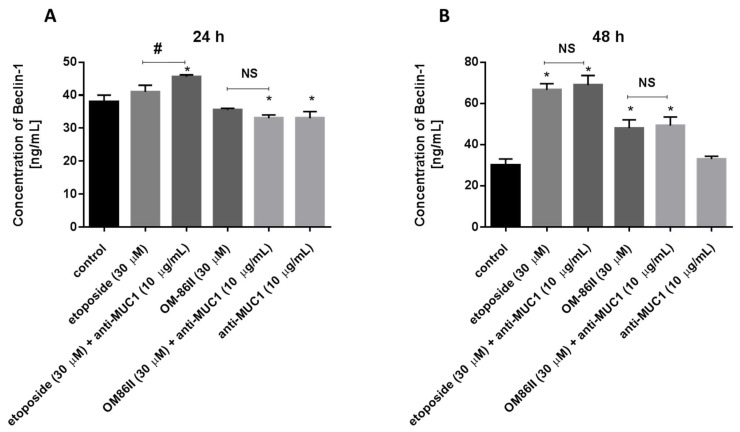
The concentration of Beclin-1 in human gastric cancer AGS cells after 24 h (**A**) and 48 h (**B**) of incubation with anti-MUC1 (10 μg/mL), OM-86II (30 μM), OM-86II + anti-MUC1 (30 μM + 10 μg/mL), etoposide (30 μM) and etoposide + anti-MUC1 (30 μM + 10 μg/mL). Data are presented in ng/mL. * *p* < 0.05 vs. control group; # *p* < 0.05. MUC1, mucin-1; OM-86II, octahydropyrazin[2,1-a:5,4-a′]diisoquinoline derivative; NS, not significant.

We checked the Beclin-1 concentration after 24 and 48 h of incubation with the analyzed compounds (Figure 14). We proved that longer exposition of cells to the combination of compounds (etoposide + anti-MUC1, OM-86II + anti-MUC1) led to the increase of the Beclin-1 concentration. The concentrations of Beclin-1 were 68.86 ng/mL for etoposide with the anti-MUC1 antibody and 49.2 ng/mL for OM-86II with the anti-MUC1 antibody after 48 h of incubation, in comparison with untreated cells (30 ng/mL).

### 2.7. Novel Diisoquinoline Derivative with Anti-MUC1 Antibody Decreased Cathepsin B Concentration in Human Gastric Cancer Cells

**Figure 15:**
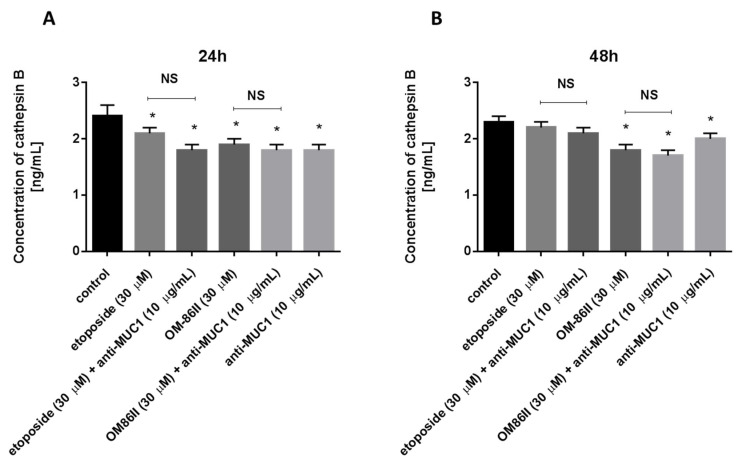
The concentration of cathepsin B in human gastric cancer AGS cells after 24 h (**A**) and 48 h (**B**) of incubation with anti-MUC1 (10 μg/mL), OM-86II (30 μM), OM-86II + anti-MUC1 (30 μM + 10 μg/mL), etoposide (30 μM) and etoposide + anti-MUC1 (30 μM + 10 μg/mL). Data are presented in ng/mL. * *p* < 0.05 vs. control group; MUC1, mucin-1; OM-86II, octahydropyrazin[2,1-a:5,4-a′]diisoquinoline derivative; NS, not significant.

Finally, the effect of the tested compounds on cathepsin B concentration is demonstrated in Figure 15. All the tested agents used alone and in combination with the anti-MUC1 antibody led to a decrease in cathepsin B concentration in comparison with the control after 24 and 48 h of incubation. The most significant effect was observed after 48 h of incubation with anti-MUC1 and OM-86II, where the cathepsin B concentration was 1.7 ng/mL.

## 3. Discussion

In recent years, meaningful progress has been observed in the treatment of gastric cancer (GC). The most standard methods to combat GC still include surgical resection, chemotherapy and radiotherapy, but it is most common to use a combination of immunotherapy, neoadjuvant chemoradiotherapy and targeted therapy in advanced gastric cancer [30]. The dysfunction of molecular signaling pathways is associated with the pathogenesis of gastric cancer. Targeted agents have been investigated for GC treatment, such as: HER-2, EGFR, VEGF/VEGFR, PI3K/mTOR, PARP, MMPs inhibitors and agents that could induce tumor cell apoptosis [31]. However, researchers are still searching for molecular targets, that may be the main points of the targeted therapy. Much interest was placed on the role of MUC1 in gastric cancer development and progression. Many reports showed its higher expression in gastric carcinoma in comparison with normal gastric mucosa, as well as a significant correlation between its abnormal expression and poor outcome [32,33,34]. Preclinical and clinical studies based on MUC1-targeted therapy are still ongoing. Monoclonal antibodies, immunoconjugates, small-molecule peptides and vaccines are in various stages of research. Combinations of a monoclonal antibody with chemotherapeutic agents might be a promising strategy in the treatment of GC.

Recently, novel diisoquinoline derivatives have been synthesized by our research group, and their anticancer potential in MCF-7 and MDA-MB-231 breast cancer cells was proven [35]. Their mechanism of action is associated with the inhibition of topoisomerase II [36,37]. We chose one of the most promising agents (OM-86II) and we tested the combination of OM-86II together with the monoclonal anti-MUC1 antibody in the treatment of AGS cancer cells. We demonstrated that such a strategy led to the induction of extrinsic and intrinsic apoptotic pathways. We observed the highest percentage of AGS gastric cancer cells with active caspase-8 and caspase-9. Caspases are proteases, which are engaged in programmed cell death and processes of inflammation. Caspase-8 and caspase-9 belong to the initiator group, whereas caspase-3 is the executioner.

Small-molecule caspase activators were tested in several studies [38]. Wolan et al. analyzed the effect of compound 1541, where the direct activation of procaspase-3 was observed with its concentration of 2.4 µM [39]. Another research team tested PAC-1, which also induced procaspase-3 activation in several cancer cell lines, but not in a direct way [40,41]. Li et al. checked the anticancer potential of a recombinant adenovirus carrying immunocaspase-3 in hepatocellular carcinoma, and the results confirmed that caspase-based gene therapy might also be a promising strategy based on apoptosis as a target [42].

Autophagy represents a genetically programmed process, which plays an essential role in many diseases, including cancer [43]. Literature data show two aspects of autophagy. One theory claims that inhibition of autophagy is necessary to receive the positive effect from anticancer therapy [44]. However, some studies demonstrated that induction of autophagy may also possess a cancer-suppressive effect. It was proven that rapamycin, nilotynib, cisplatin and docetaxel were able to inhibit the proliferation of cancer cells by promoting autophagy [45,46,47]. The basis for looking for novel autophagy inducers was the fact that mice which possess the loss of one allele of the *Beclin-1* gene developed tumors [48]. Tat-Beclin-1 has been demonstrated as an autophagy activator [49]. Additionally, multiple natural products from traditional medicine, such as genipin, tanshinone IIA, liquiritin and DSGOST (Danggui-Sayuk-Ga-Osuyu-Saenggang-Tang), exert the ability to induce autophagy. Genipin has shown anticancer properties and induces both apoptosis and autophagy. It was proven that it improved the sensitivity of AGS gastric cancer cells to oxaliplatin. Tanshinone IIA together with adriamycin also triggered apoptosis and autophagy in gastric cancer cells. Moreover, liquiritin is responsible for increasing Beclin-1 expression and the activation of apoptosis and autophagy. Finally, DSGOST enhanced the sensitivity of gastric cancer cells to cisplatin and triggered apoptotic and autophagic cell death [50].

The results from our study showed different effects depending on time of incubation with the tested compounds. After 24 h of incubation, we demonstrated that none of the tested compounds induced autophagy. The longer exposition (48 h) to the tested compounds resulted in an increase of LC3A, LC3B and Beclin-1 concentrations. Upon treatment with a combination of etoposide or OM-86II with anti-MUC1, we found that they induced autophagy in gastric cancer cells, in comparison with untreated samples.

MUC1 promotes proliferation and invasion of cancer cells through the activation of the PI3K/Akt/mTOR and ERK1/ERK2 pathways [51]. The activation of Akt leads to the inhibition of apoptosis [6]. We demonstrated that the combination of the novel diisoquinoline derivative with the anti-MUC1 antibody decreased the concentration of the final point of the PI3K/Akt pathway (mTOR) after 24 and 48 h of incubation. The FDA approved the proteasome inhibitor known as bortezomid for multiple myeloma and cell lymphoma, and it also exerted anticancer potential in gastric cancer cells and is now being tested in combination with chemotherapeutic agents. Its mechanism of action is associated with the inhibition of the NF-𝜅B, ERK1/ERK1 and Akt signaling pathways.

The interaction of MUC1 and ICAM1 promotes the migration of cancer cells and increases the invasive potential of tumor cells [52]. We demonstrated that all of the tested agents decreased sICAM1 concentration, but the most effective one was the combination of the novel diisoquinoline derivative with the anti-MUC1 antibody. Our tested treatment inhibited the ability of cancer cells to migrate by decreasing the sICAM1 concentration.

In the literature, an interaction between matrix metalloproteinases (MMPs) and MUC1 has not been found yet, but abnormal synthesis of MMPs is associated with a poor prognosis of patients with gastric cancer. Researchers are still looking for novel MMPs inhibitors. Marimastat is a representant of MMP inhibitors and its anticancer efficiency was shown in gastric cancer patients [53]. We proved that our tested combination of OM-86II with the anti-MUC1 antibody reduced only MMP-9, but not MMP-2 concentration in AGS gastric cancer cells.

Many reports underlined the uncontested role of cathepsin B in cancer progression [54,55,56]. It is overexpressed in many tumors, especially in gastric cancer cells, and it might be a potential therapeutic target [57]. We demonstrated that the combination of the novel diisoquinoline derivative with the anti-MUC1 antibody reduced the concentration of cathepsin B after 24 and 48 h of incubation. Tummalapalli et al. showed that downregulation of cathepsin B and MMP-9 led to a decrease of growth and invasion in a meningioma cell line [58].

## 4. Materials and Methods

### 4.1. Compounds

The novel diisoquinoline derivative (OM-86II) was synthesized using previously standardized methods [59,60].

### 4.2. Cell Culture

AGS-CRL-1739 human gastric cells were obtained from ATCC—American Type Culture Collection (Manassas, VA, USA). Cells were cultured in DMEM (Dulbecco′s Modified Eagle Medium), which was supplemented with 10% fetal bovine serum (FBS) and a 1% cocktail of penicillin and streptomycin. Cells were seeded in Costar flasks and grown in 5% CO_2_ at 37 °C to reach about 90–95% of subconfluency. Then, human gastric cancer cells were treated with 0.05% trypsin and 0.02% EDTA (ethylenediaminetetraacetic acid) in calcium-free phosphate buffered saline, counted in a hemocytometer and seeded at 5 × 105 cells/well in 6-well plates (Nunc) in 2 mL of growth medium (DMEM). Cells that reached about 80% of confluency were used for further analysis.

### 4.3. Cell Viability Assay

AGS-CRL-1739 cells were seeded in six-well plates (1 × 10^6^) and cultured as described above. Cells were incubated for 24 and 48 h with different doses of the tested compounds used alone (anti-MUC1, etoposide, OM-86II) and in combination with the anti-MUC1 antibody (anti-MUC1 + etoposide, anti-MUC1 + OM86II). The concentration of the anti-MUC1 antibody was 10 μg/mL. The concentration of etoposide and OM-86II was 30 μM. The doses of compounds were chosen after the preliminary experiment and the tested concentrations were lower than IC_50_ values. MTT (3-(4,5-dimethylthiazole-2-yl)-2,5-diphenyltetrazolium bromide) was used as a substrate of reaction and the absorbance of the converted dye in live cells was measured at a wavelength of 570 nm [61]. Cell viability of the gastric cancer cells in the presence of the analyzed agents was calculated as a percent of control cells.

### 4.4. [^3^H]thymidine Incorporation Assay

The effect of the studied compounds: etoposide, OM-86II and the anti-MUC1 antibody, as well as the combination of etoposide or OM-86II with the anti-MUC1 antibody, on cells’ proliferation was also tested. AGS-CRL-1739 cells were seeded in six-well plates and cultured as described above. Cells were treated with different concentrations of the tested compounds and 0.5 μCi of [^3^H]thymidine for 24 and 48 h at 37 °C. The cells were harvested by trypsinization and washed several times in the cold PBS (phosphate-buffered saline) (10 min/1.500 g) until the dpm in the washes were similar to the reagent control. Radioactivity was determined by liquid scintillation counting. [^3^H]thymidine uptake was expressed as dpm/well.

### 4.5. Dual Acridine Orange/Ethidium Bromide Fluorescent Staining

Dual acridine orange/ethidium bromide fluorescent staining was performed to confirm the effect of the tested compounds on programmed cell death and then visualized under a fluorescent microscope, Nikon Eclipse Ti, with an inverted camera (Nikon Instruments Inc., Melville, NY, USA). AGS-CRL-1739 human gastric cancer cells were exposed to different concentrations of the tested compounds, such as: etoposide, OM-86II and the anti-MUC1 antibody, as well as combinations of etoposide or OM-86II with the anti-MUC1 antibody for 24 and 48 h. The cell suspension (250 µL) was stained with 10 µL of the dye mixture (10 µM acridine orange and 10 µM ethidium bromide), which was prepared in PBS. Cells cultured in a drug-free medium were used as controls. The morphology of two hundred cells per sample was carried out by fluorescent microscopy within 20 min. The obtained results were analyzed with NIS-Elements software (Nikon Instruments Inc., Melville, NY, USA) [36].

### 4.6. Determination of Mitochondrial Membrane Potential

The lipophilic cationic probe 5,5′,6,6′-tetrachloro-1,1′,3,3′-tetraethylbenzimidazolcarbocyanine iodide (JC-1 Mitoscreen kit; BD Biosciences, San Jose, CA, USA) was used to check the disruption of the mitochondrial membrane potential (MMP) [62]. Briefly, unfixed cells were washed and resuspended in PBS containing 10 mg/mL of JC-1. AGS cells were then incubated for 15 min at room temperature in the dark, washed, and resuspended in PBS for immediate BD FACSCanto II flow cytometry analysis. The percentage of cells with disrupted MMP was calculated in the FACSDiva software (both from BD Bioscences Systems, San Jose, CA, USA).

### 4.7. Caspase-8 Enzymatic Activity Assay

The FAM-FLICA Caspase-8 Kit (ImmunoChemistry Technologies, Bloomington, MN, USA) was used to determine caspase-8 activity after treatment of AGS-CRL-1739 cells with etoposide (30 μM), OM-86II (30 μM) and the anti-MUC1 antibody (10 μg/mL), as well as a combination of etoposide (30 μM) or OM-86II (30 μM) with the anti-MUC1 antibody (10 μg/mL) for 24 and 48 h. After incubation, cells were harvested and washed with cold buffer PBS. Then, 5 μL of diluted FLICA reagent and 2 μL of Hoechst 33,342 were added to 93 μL of cell suspension and mixed by pipetting. The gastric cancer cells were incubated for 60 min at 37 °C. After that time, the cells were washed twice in 400 μL of apoptosis wash buffer and centrifuged at 300× *g*. After the last wash, the cells were resuspended in 100 μL of apoptosis wash buffer and supplemented with 10 μg/mL of PI. Analysis was performed using the BD FACSCanto II flow cytometer, and the obtained results of the study were analyzed with FACSDiva software (both from BD Biosciences Systems, San Jose, CA, USA) [63].

### 4.8. Caspase-9 Enzymatic Activity Assay

The FAM-FLICA Caspase-9 Kit (ImmunoChemistry Technologies, Bloomington, MN, USA) was used to analyze caspase-9 activity, and it was measured according to the manufacturer’s instructions. The AGS-CRL-1739 cells were exposed to etoposide (30 μM), OM-86II (30 μM) and the anti-MUC1 antibody (10 μg/mL), as well as a combination of etoposide (30 μM) or OM-86II (30 μM) with the anti-MUC1 antibody (10 μg/mL) for 24 and 48 h. After that, cells were harvested and washed with cold buffer PBS. FLICA reagent (5 μL) and Hoechst 33,342 (2 μL) were added to a cell suspension (93 μL) and mixed by pipetting. The cells were incubated for 60 min at 37 °C and then washed twice using a wash buffer and centrifuged at 300× *g*. After the last wash, cells were resuspended in 100 μL of apoptosis wash buffer and supplemented with 10 μg/mL of PI. The BD FACSCanto II flow cytometer was used to perform the analysis of the results [63].

### 4.9. Determination of mTOR, sICAM1, MMP-2, MMP-9, Bax, LC3A, LC3B, Beclin-1 and Cathepsin B

High-sensitivity assay kits (EIAab Science Co., Ltd., Wuhan, China) were used to determine the concentrations of proteins in supernatants or cell lysates from AGS cell culture after 24 and 48 h of incubation with the tested compounds: etoposide (30 μM), OM-86II (30 μM) and the anti-MUC1 antibody (10 μg/mL), as well as a combination of etoposide (30 μM) or OM-86II (30 μM) with the anti-MUC1 antibody (10 μg/mL). The microtiter plate provided in this kit has been pre-coated with an antibody specific to the analyzed antigen. Standards and samples were added to the appropriate microtiter plate wells. After 2 h of incubation at 37 °C, the plate was incubated with a biotin-conjugated antibody for 1 h at 37 °C. Then, microplate wells were aspirated and washed three times and then incubated with avidin conjugated to Horseradish Peroxidase (HRP). Then, a TMB (3,3′,5,5′-tetramethylbenzidine) substrate solution was added to each well. Those wells that contained the target antigen exhibited a change in color. The enzyme–substrate reaction was terminated by the addition of a sulfuric acid solution and the color change was measured spectrophotometrically at a wavelength of 450 ± 2 nm. The antigen concentration in the samples was determined by comparing the O.D. of the samples to the standard curve [60].

### 4.10. Statistical Analysis

The results of the study are presented as mean ± standard deviation (SD) from three independent experiments. The statistical analysis was performed using GraphPad Prism Version 6.0 (GraphPad Software, Inc., San Diego, CA, USA). ANOVA and Tukey tests were used to demonstrate the differences between the control cells and the cells exposed to varying concentrations of the tested compounds. A statistically significant difference was defined at *p* < 0.05.

## 5. Conclusions

**Figure 16:**
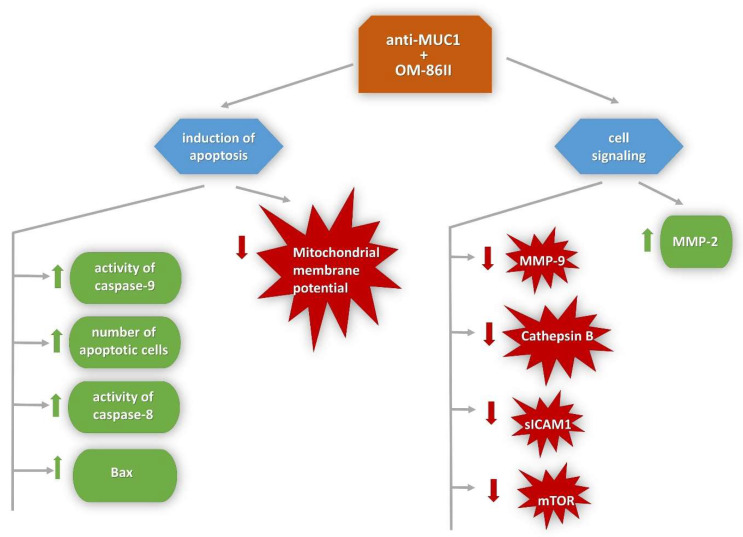
Mechanism of anticancer action of the anti-MUC1 antibody used with the novel diisoquinoline derivative in human gastric cancer cells.

The findings of this study proved that the anti-MUC1 antibody contributed to the increased sensitivity of gastric cancer cells to the novel diisoquinoline derivative (OM-86II). Its mechanism of action is pleiotropic, and it is based on the induction of extrinsic and intrinsic apoptotic pathways, a decrease of mitochondrial membrane potential as well as inhibition of proteins responsible for increased proliferation and invasion of gastric cancer cells with overexpressed MUC1, such as mTOR, sICAM1 and MMP-9 (Figure 16). Its increased cytotoxicity was also associated with autophagy induction, which correlated with the increased concentration of LC3A, LC3B and Beclin-1 after 48 h of incubation. Our studies confirmed that the combination of the chemotherapeutic agent with the monoclonal antibody against MUC1 could be a promising strategy in gastric cancer treatment with aberrant expression of MUC1, but further in vivo examinations will be required.

## Data Availability

The datasets used and/or analyzed during the current study are available from the corresponding author on reasonable request.

## Sample Availability

Samples of the compounds are not available from the authors.
